# The state of food composition databases: data attributes and FAIR data harmonization in the era of digital innovation

**DOI:** 10.3389/fnut.2025.1552367

**Published:** 2025-03-19

**Authors:** Sarah Brinkley, Jenny J. Gallo-Franco, Natalia Vázquez-Manjarrez, Juliana Chaura, Naa K. A. Quartey, Sahar B. Toulabi, Melanie T. Odenkirk, Eva Jermendi, Marie-Angélique Laporte, Herman E. Lutterodt, Reginald A. Annan, Mariana Barboza, Endale Amare, Warangkana Srichamnong, Andres Jaramillo-Botero, Gina Kennedy, Jaclyn Bertoldo, Jessica E. Prenni, Maya Rajasekharan, John de la Parra, Selena Ahmed

**Affiliations:** ^1^The Periodic Table of Food Initiative, Bioversity International, CGIAR, Rome, Italy; ^2^The Periodic Table of Food Initiative, The International Center for Tropical Agriculture (CIAT), CGIAR, Cali, Colombia; ^3^Instituto Nacional de Ciencias Médicas y Nutrición Salvador Zubirán, Mexico City, Mexico; ^4^iOMICAS Research Institute, Pontificia Universidad Javeriana Cali, Cali, Colombia; ^5^Department of Biochemistry and Biotechnology and Department of Food Science and Technology, College of Science Kwame Nkrumah University of Science and Technology - KNUST, Kumasi, Ghana; ^6^Department of Horticulture and Landscape Architecture, Colorado State University, Fort Collins, CO, United States; ^7^Division of Human Nutrition and Health, Wageningen University and Research, Wageningen, Netherlands; ^8^The Periodic Table of Food Initiative, Bioversity International, Montpellier, France; ^9^Genome Center and Innovation Institute for Food and Health, University of California Davis, Davis, CA, United States; ^10^Nutrition, Environmental Health and Non-Communicable Diseases Research Directorate, Ethiopian Public Health Institute, Addis Ababa, Ethiopia; ^11^Institute of Nutrition, Mahidol University, Salaya, Thailand; ^12^Chemistry and Chemical Engineering, California Institute of Technology, Pasadena, CA, United States; ^13^The Periodic Table of Food Initiative, American Heart Association, Dallas, TX, United States; ^14^The Rockefeller Foundation, New York, NY, United States

**Keywords:** food composition database, food composition data management, food composition data, food quality, metadata, food components, FAIR data, nutritional database

## Abstract

**Introduction:**

Food composition databases (FCDBs) are essential resources for characterizing, documenting, and advancing scientific understanding of food quality across the entire spectrum of edible biodiversity. This knowledge supports a wide range of applications with societal impact spanning the global food system. To maximize the utility of food composition data, FCDBs must adhere to criteria such as validated analytical methods, high-resolution metadata, and FAIR Data Principles (Findable, Accessible, Interoperable, and Reusable). However, complexity and variability in food data pose significant challenges to meeting these standards.

**Methods:**

In this study, we conducted an integrative review of 35 data attributes across 101 FCDBs from 110 countries. The data attributes were categorized into three groups: general database information, foods and components, and FAIRness.

**Results:**

Our findings reveal evaluated databases show substantial variability in scope and content, with the number of foods and components ranging from few to thousands. FCDBs with the highest numbers of food samples (≥1,102) and components (≥244) tend to rely on secondary data sourced from scientific articles or other FCDBs. In contrast, databases with fewer food samples and components predominantly feature primary analytical data generated in-house. Notably, only one-third of FCDBs reported data on more than 100 food components. FCDBs were infrequently updated, with web-based interfaces being updated more frequently than static tables. When assessed for FAIR compliance, all FCDBs met the criteria for Findability. However, aggregated scores for Accessibility, Interoperability, and Reusability for the reviewed FCDBs were 30, 69, and 43%, respectively.

**Discussion:**

These scores reflect limitations in inadequate metadata, lack of scientific naming, and unclear data reuse notices. Notably, these results are associated with country economic classification, as databases from high-income countries showed greater inclusion of primary data, web-based interfaces, more regular updates, and strong adherence to FAIR principles. Our integrative review presents the current state of FCDBs highlighting emerging opportunities and recommendations. By fostering a deeper understanding of food composition, diverse stakeholders across food systems will be better equipped to address societal challenges, leveraging data-driven solutions to support human and planetary health.

## Graphical abstract

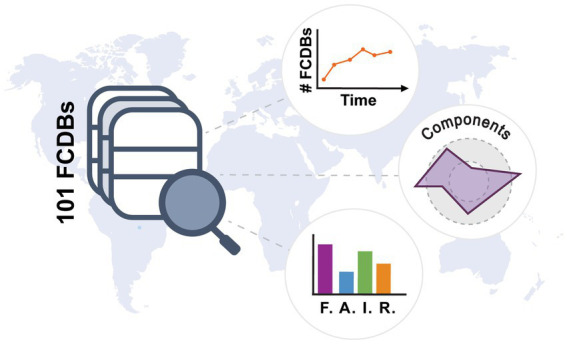

## 1 Introduction

Food composition data are essential for informing solutions to today’s human and planetary health challenges including loss of biodiversity, food insecurity, and diet-related chronic disease (1–3). Food composition databases (FCDBs) are foundational tools across sectors, including agriculture, food science, nutrition, public health, and policymaking, supporting crop breeding, product development, nutritional assessments, and public health initiatives. By advancing the availability and accessibility of FCDBs it is possible to promote evidence-based solutions to harness the power of food to foster well-being and sustainable practices across food systems. Contributors and curators of FCDBs perform a critical role in providing access to and enabling the use of reliable, high-quality data on food and food composition (4–6). To maximize the utility of data from FCDBs for diverse applications, these databases should meet three key criteria: (i) the utilization of validated methods and computational approaches to ensure data accuracy and consistency (1) (ii) the inclusion of detailed, high-resolution metadata that provide essential context about the source, preparation, and analysis of foods (7), and (iii) adherence to FAIR Guiding Principles for data management and stewardship, making data Findable, Accessible, Interoperable, and Reusable (FAIR), to facilitate integration, sharing, and practical application across sectors (8).

Given the importance of FCDBs, conducting an integrative review of their current state was timely. Here, we evaluated FCDBs spanning multiple countries worldwide based on 35 data attributes, emphasizing the range of foods and components included, data harmonization, and adherence to FAIR data governance principles. We also present an overview of the inception and historical evolution of these databases, highlighting their role in enabling researchers to monitor trends in food crop variation, particularly in response to climate change and biodiversity loss. Additionally, we examined how the data are presented and accessed from the end user’s perspective, including researchers, policymakers, and food systems stakeholders, ensuring accessibility and usability across sectors. Finally, we assessed the compliance of the analyzed databases with the FAIR data criteria—Findable, Accessible, Interoperable, and Reusable. The results of this evaluation provide a detailed snapshot of the current state of FCDBs and identify key opportunities to enhance their functionality as dynamic and integrative resources. These enhancements can foster cross-sectoral collaboration and drive innovative solutions. Strengthening FCDBs will unlock their potential to support global efforts in preserving food biodiversity, addressing nutrition insecurity, and mitigating diet-related chronic diseases through evidence-based strategies.

## 2 Background

In their first iterations in the 1800s, food composition data were compiled in Food Composition Tables (FCT) that strictly focused on proximate composition (e.g., carbohydrates, fat, protein, moisture, and ash) of a limited cross-section of foods in a “typical” (i.e., Western-leaning) diet (9, 10). In contrast, modern FCDBs are characterized by a high degree of heterogeneity encompassing a diverse range of data sources, analytical methods, nomenclature and terminologies, food types, data processing methods, data formats, and overall relevance to various audiences (11). This diversity reflects advancements in analytical technologies, which have expanded the scope of nutritional data to include foodomics-level insights such as the thousands of specialized metabolites in foods including bioactive polyphenols, sterols, terpenes, and carotenoids (1, 12, 13). However, despite these advancements, foodomics data remain underrepresented in FCDBs, and the relationship of the thousands of specialized metabolites to adequate nutrition and health is still being established.

Efforts to address the variability and gaps in FCDBs have been ongoing for decades. European compilers at the International Network of Food Data Systems (INFOODS) administered by the Food and Agriculture Organization (FAO) of the United Nations, the EU Network of Excellence: European Food Information Resource (EuroFIR), and others have long recognized the need for and led efforts on the harmonization of food composition data (14–16). Since their inception, these international efforts have advanced food composition database management by promoting the inclusion of mandatory metadata thesauri, standardized analytical methods (e.g., AOAC), food and food component nomenclature, and methods of conversion (17).

Despite these advances, national FCDBs, which track the nutrient composition of foods based on dietary intake patterns at the national level, often reflect regional biases. For instance, the United States Department of Agriculture’s FoodData Central (FDC) (18) widely recognized as the gold standard in food composition data, is a crucial resource for aggregating food data which shapes the U.S. national nutrition guidelines and associated food and nutrition policies (10). However, with a federal mandate to survey the nation’s most widely consumed foods, FDC may still have sparse coverage of foods found in regionally distinct diets (19). For example, Lozano et al. (20), report 97 commonly consumed foods of Hawaii, like taro-based poi or pohole (i.e., fiddlehead fern or *Diplazium esculentum*) are not represented in FDC’s Food and Nutrient Database for Dietary Studies (FNDDS). This paucity of food representation leaves nutrition professionals to rely on closely related food analogs which may result in dietary assessment error disproportionately impacting the health outcomes of the populations who depend on these foods (20, 21).

While efforts to increase the edible biodiversity represented in global food composition databases exist (15), there is still a panoply of edible species yet to be characterized (22–24). To overcome the disparity of underrepresentation, national FCDBs must be enriched with data on regionally distinct staples and less utilized, culturally significant foods, for example, edible insects like house cricket (*Acheta domesticus*) and dung beetle (*Paragymnopleurus aethiops*) in Thailand, African palm weevil (*Rhynchophorus phoenicis*) in Ghana (25–27), and *Amaranthus* spp. endemic throughout sub-Saharan Africa and the Americas (28). The characterization of traditional foods, like amaranth or nopal (*Opuntia ficus-indica*) from Mexico and other regions, will allow for further safeguarding of traditional knowledge while integrating nutrient-dense ingredients with high potential for reducing noncommunicable disease (28, 29) into regional and global nutrition frameworks. Inclusion of these foods is crucial for accurately reflecting true biodiversity and addressing food security challenges. For instance, the moriche palm (*Mauritia flexuosa*) serves as a vital resource in Colombia, providing not only nutritious fruits rich in vitamins A and E for traditional dishes but also providing materials for crafts and construction, thus supporting local economies and cultural practices (30). Expanding the characterization of the world’s edible biodiversity will not only reduce assessment error and improve cross-cultural relevance (31), greater understanding of chemodiversity will also inspire a cornucopia of future foods (23).

Secondary data (i.e., food composition data from another FCDB, peer-reviewed manuscript, or another external source) may also lead to data homogenization or inaccurate representations of the local food supply. Due to resource constraints, national FCDBs often rely on the primary data generated by the USDA or other literature-reported primary food composition data. Primary data refer to food composition data derived from in-house, laboratory analysis, which is generated specifically for the purpose of compiling the FCDB. Databases may recycle primary data directly, use methods of conversion, or publish an amalgamation of both primary and secondary data. While the use of secondary data facilitates faster data compilation there are often challenges in harmonizing analytical methodologies, conversion factors, and other technical aspects related to data processing and reporting (11, 32). Additionally, the nutrient content of some foods can vary significantly between countries and regions due to factors such as genetics (i.e., cultivars, variety, or breed), environment (i.e., climate, soil, geography, and biotic and abiotic factors), and agricultural management, not to mention postharvest and processing factors (10, 33).

Building on these advancements, modern FCDBs are presented with an opportunity to adopt better data governance and stewardship principles. International quality standards such as the FAIR Data Guiding Principles (34) promote the inclusion of metadata and ontologies (35) to make food composition data more discoverable, shareable, usable, and citable. Originally, FAIR Data Principles were created to increase the exchange of scholarly data products (34), but by extension, the utility of FAIRness for food composition data management and stewardship facilitates the sharing of knowledge on foods and food composition (36). FCDBs with harmonized food composition data support international research and policy-making, including addressing cross-border nutritional challenges and promoting a transnational understanding of the world’s food supply with increasingly interconnected food systems (37). With this unified approach, there is potential for a greater comparative understanding of nutritional resources globally, yet data harmonization should not result in data homogenization.

## 3 Materials and methods

### 3.1 Assessing the landscape of food composition databases

We conducted an integrative review of food composition databases. The systematic approach of the integrative review followed a rigorous systematic literature review methodology but integrated diverse sources of research (i.e., disparate food composition databases) (38, 39). We began our investigation with a broad research question aimed at assessing the current landscape of food composition databases globally: “What are the gaps and opportunities in food composition analysis and data collection in an era of digital innovation”? We then conducted multiple searches using Google Search and Google Scholar in private browser tabs using the keywords “nutritional database” OR “food composition database”. The search process took place between April and December 2023 with all queries performed in English. The locations where the searches were conducted were globally distributed between Europe, North America, and South America. All the results were reviewed. A minimum of two researchers independently conducted each search for food composition databases.

The search results were carefully reviewed to identify national and international FCDBs, foodomics databases, and other nutritional databases for inclusion in our integrative review. The search uncovered resources dating back to the 1950s, which informed our decision to include databases from 1950 to 2024. Our search additionally revealed food composition database repositories (i.e., collections of food composition databases) from authoritative sources essential for research and policymaking. In addition to individual FCDBs, we also included resources previously compiled by such authoritative sources including FAO/INFOODS (40), EuroFIR (14), Danish Food Informatics (16), and the World Nutrient Databases for Dietary Studies (WNDDS) (41). The integration of FCDBs from these open-access repositories enhanced the accuracy and scope of this integrative review, enabling more robust analyses and comparisons.

**Figure 1:**
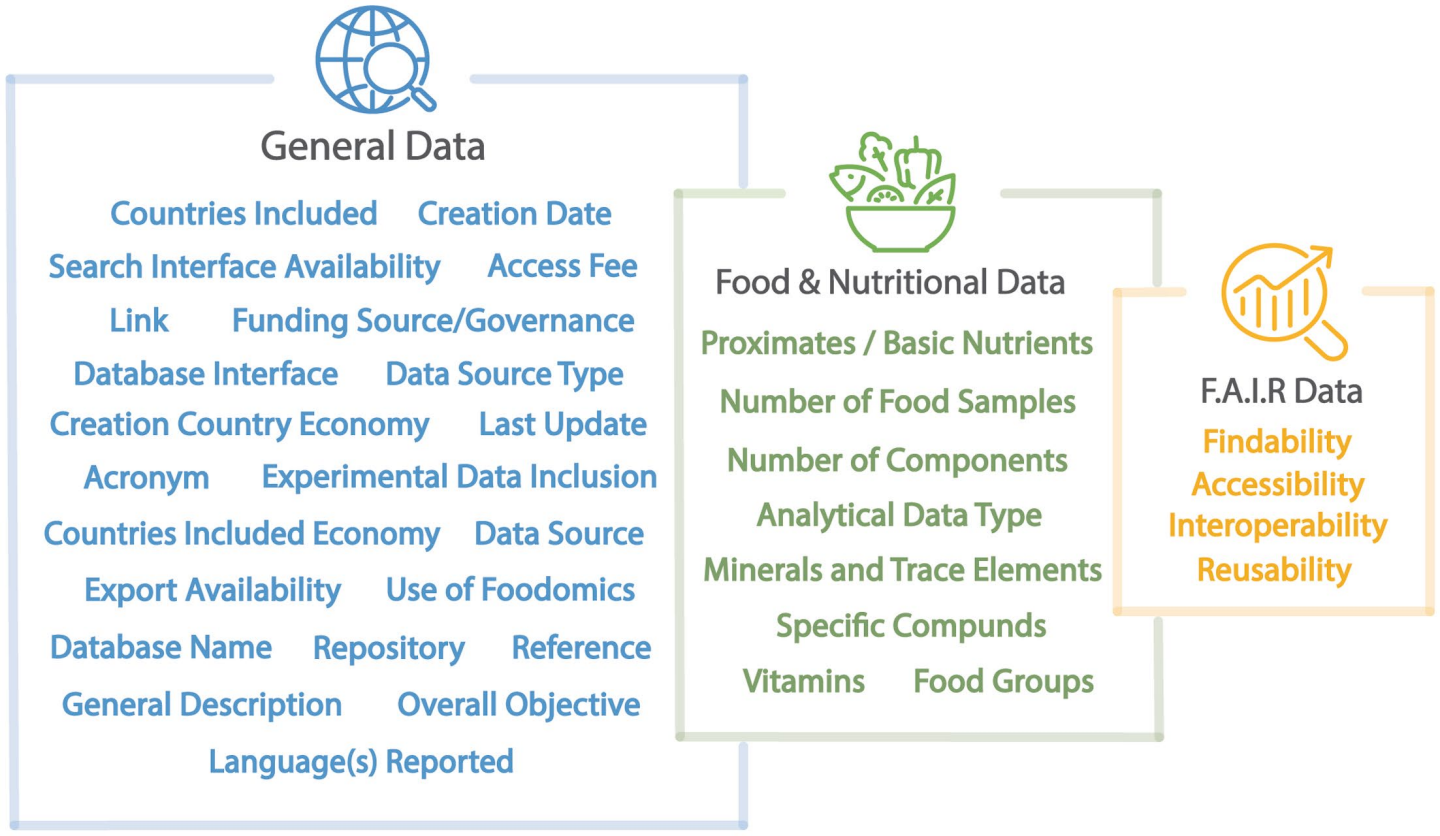
The data attributes used to catalog the food composition database characteristics. For the full descriptions of the 35 attributes used and the data collected, refer to Annex 1 and Supplementary Tables S1–S5.

After the systematic search, teams of reviewers were assembled to conduct quality appraisal and data extraction steps. A set of 35 data attributes was established (Figure 1) to characterize the identified FCDBs. Multidisciplinary teams of 16 researchers from eight countries, with expertise in food science, nutrition, public health, agricultural sciences, analytical chemistry, biology, biotechnology, bioinformatics, and computer science were assembled to define FCDBs characteristics and extract data on the 35 data attributes used in the review. The transdisciplinary researchers were from multiple geographies and cultural-linguistic backgrounds, and thus, they reviewed databases in Dutch, English, French, Hungarian, Italian, and Portuguese in their original language. For databases available in languages other than these languages, researchers used Google Translate to translate the necessary information for data extraction. All the FCDBs were distributed in random order for data extraction by the transdisciplinary research teams. Teams of two reviewers conducted a thorough and independent review and quality appraisal of each food composition database to decide upon inclusion or exclusion. Finally, a third round of revisions was carried out to resolve any discrepancies identified by the first two reviewers and to ensure consistency and data quality across the entire dataset. A smaller group of researchers was selected to conduct a third and final review.

The 35 attributes were categorized into three groups: general information about the database (23 attributes), food and nutrient data (8 attributes), and FAIR Data Principles (4 attributes evaluated against 13 criteria).

The general database attributes included: Database name, Acronym, Link, Reference, Related repository, Funding source/Governance, Country of creation, Creation Country Economy Classification (WBA), Countries included, Economy classification of countries included (WBA), Creation date, Last update, Reported languages, Data source type, Database interface, Export availability, Access fee, Foodomics data availability, Experimental data inclusion, Search interface, Data source, Overall objective, and General description. For definitions of the data attributes, refer to the Readme file in Annex 1 of the Supplementary materials. For the economic classification of countries, we applied the World Bank economic classification system (42). To simplify interpretations, we grouped countries classified as High-Income together with those categorized as Upper-Middle-Income, and countries classified as Low-Income together with those categorized as Lower-Middle-Income.

The food and nutrient data attributes included: the number of food samples (i.e., total number of food samples including diverse food types such as multi-ingredient foods), food groups, number of components, data type, proximate composition, minerals and trace elements, vitamins, and specific compounds. Due to the lack of standardization in food-specific metadata, we evaluated food coverage across 13 predefined food groups, based on the methodology of Jarvis et al. (23). These groups included: (i) algae, (ii) mushrooms, (iii) herbs and spices, (iv) oily plants, (v) beverages, (vi) nuts and seeds, (vii) processed foods, (viii) beans and pulses, (ix) fruits, (x) terrestrial animal products, (xi) vegetables, (xii) aquatic animal products, and (xiii) cereals and grains. Notably, processed foods were an additional category not included in Jarvis et al. (23). Processed foods are defined as any foods that are not in a raw or minimally processed state.

Criteria were established to determine if a database should be included or excluded from this integrative review: (i) problems accessing the database, (ii) the absence of food composition data, (iii) the presence of only one metabolite unrelated to its presence in foods, and (iv) repositories or lists of databases.

### 3.2 Data stewardship and FAIR data guiding principles

To assess data stewardship best practices, all FCDBs underwent an evaluation of FAIR Data Guiding Principles, which included assessments of data Findability, Accessibility, Interoperability, and Reusability. Emphasis was placed on machine readability, as described by Wilkinson et al. (34), due to the increasing scale of big data, the advent of artificial intelligence, and the need for computational support in research. FCDBs that met the individual criteria used to assess FAIR Principles were assigned a 1. Any misalignment of FCDBs or ambiguous agreements with criteria on specific FAIR data assessments were assigned a 0 (Supplementary Tables S3, S4).

The initial step in adhering to the FAIR Data Guiding Principles involves locating the data. The findable criteria were defined as the database being easily discoverable by both humans and machine-learning interfaces. To determine if the database is findable, it must possess a globally unique, persistent identifier such as a Uniform Resource Locator (URL) or a Digital Object Identifier (DOI) (see Supplementary Table 3). In some cases, the search engines or the global databases provided broken URLs for the FCDBs, requiring extensive searches (i.e., Archive.org) to find the updated links (43).

To evaluate data accessibility, four criteria were assessed. First, FCDBs were classified as publicly accessible or available in a controlled manner for users with appropriate permissions, either for free or for a fee. Although databases were not penalized for requiring a fee, open-access was considered a positive factor in data accessibility. Second, we examined whether the database allowed data downloads, and if so, whether the output was provided in a machine-readable format (e.g., CSV, XML, JSON, and MySQL). For this integrative review, PDFs were considered non-machine readable. Finally, the presence of an application programming interface (API) was assessed as the final metric for accessibility (see Supplementary Table 3).

The interoperability of a database was assessed based on the adoption of standardized protocols and formats that enable both humans and machines to retrieve and interpret the data effectively. To evaluate interoperability, we examined whether the database employed a formal, accessible, shared, and widely applicable language for representing knowledge related to: (i) food groups, (ii) scientific names, (iii) nutritional components, and (iv) analysis methods for primary and secondary data types (see Supplementary Table 3).

Lastly, databases were considered reusable if they met the following criteria: (i) inclusion of a clear and accessible data usage license, (ii) association with detailed provenance information, and (iii) provision of metadata that comprehensively describes the context in which the data were generated, as outlined by Wilkinson et al. (34), (see Supplementary Table 3).

### 3.3 Meta-analysis and statistics

Metadata were harmonized for statistical analysis with RStudio Version 4.3.2 (R Studio, Boston, MA, United States). Statistical analysis and data visualization were performed using the following packages: R World Maps, ggplot2, maps. Code is available on GitHub at: https://github.com/scbrinkley/ptfi-fs.

## 4 Results

### 4.1 Identification and inclusion of databases

**Figure 2:**
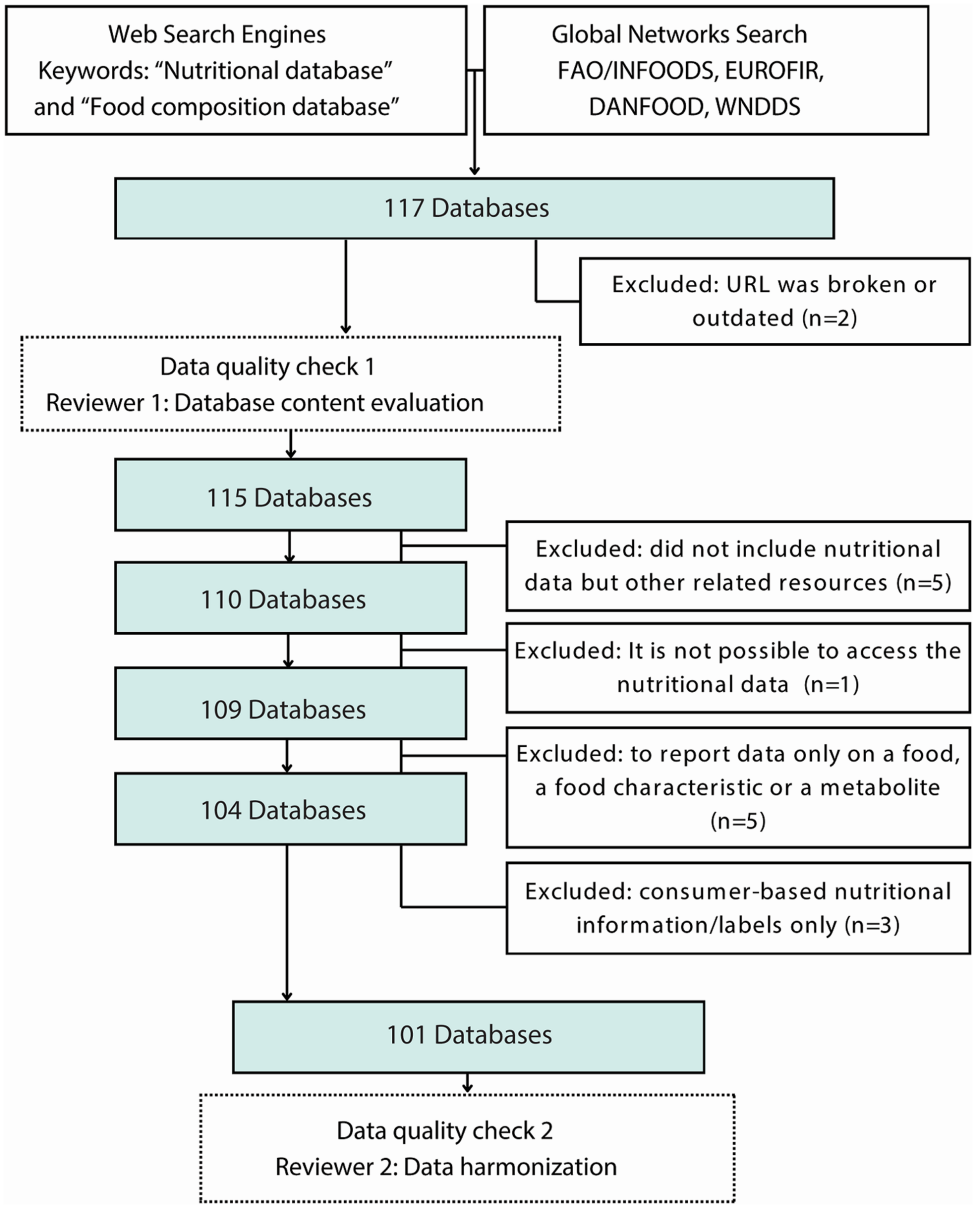
Food composition databases search, filtering, and quality appraisal protocol. All the databases were submitted to two data quality check controls to be considered in this integrative review.

A total of 117 FCDBs were compiled from web resources. A set of inclusion criteria was applied to each database to establish if it should be included in this integrative review. These criteria included: accessibility and availability of nutritional data, inclusion of comprehensive information about food components, and/or inclusion in a national or international effort to evaluate food composition. From the initial list of FCDBs, we excluded 17 databases because they did not meet the inclusion criteria (Supplementary Table 6). Specifically, these 17 FCDBs were excluded because the webpage was not available, they contained only single food components, they only contained data on food flavor, and/or they solely provided lists of other food composition databases. We arrived at a final count of 101 FCDBs (Figure 2).

### 4.2 Global overview of food composition databases

**Figure 3:**
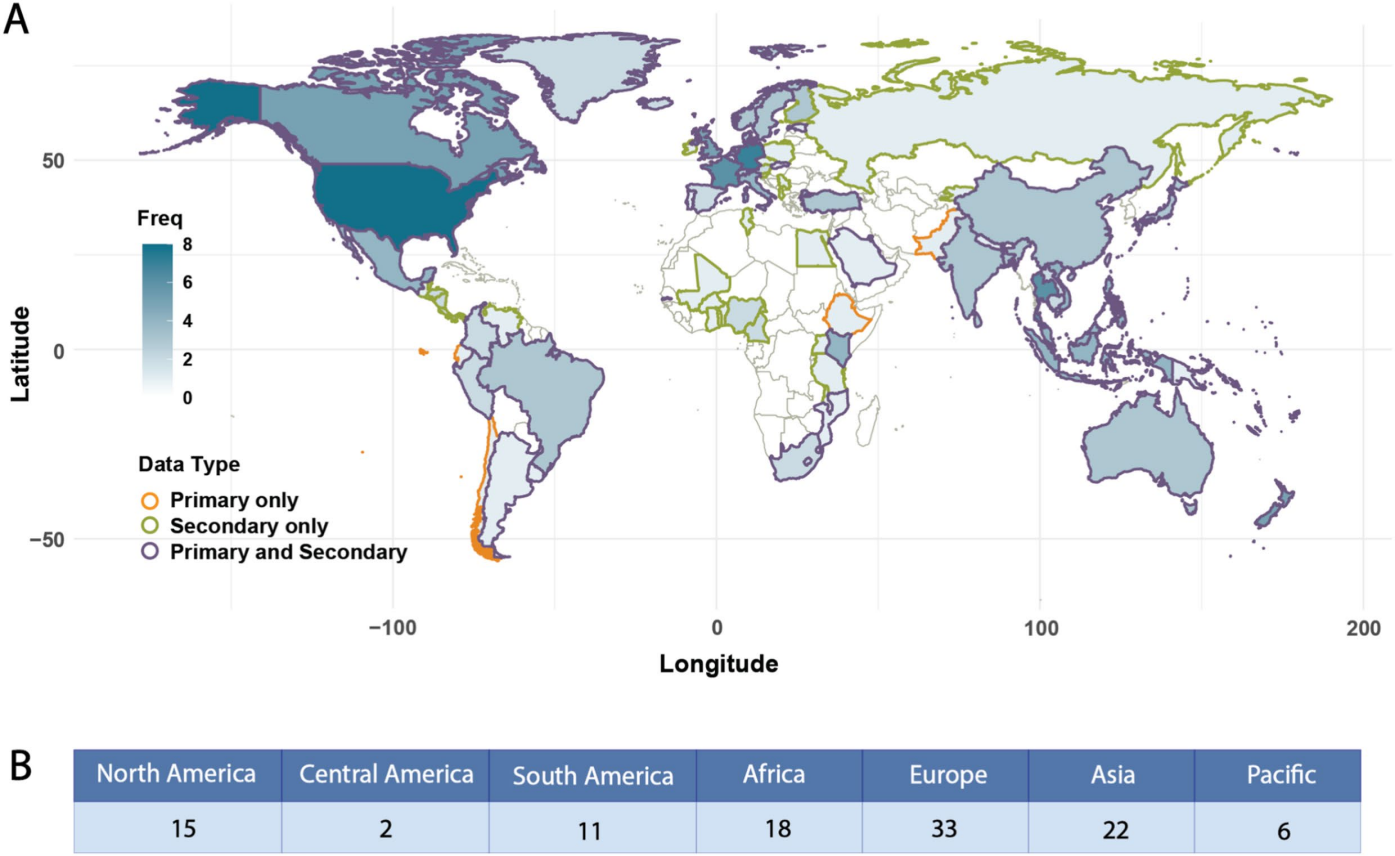
Global distribution of food composition data. **(A)** World map showing the frequency of countries’ food composition data representation across databases. *Note: the blue color gradient (0–8+) indicates the frequency of inclusion reported per country. Darker blue represents higher frequencies (8+), while lighter blue and white indicate lower frequencies (0–1). Countries are outlined in orange if they only report primary data, green if they only report secondary data, and purple if they report both primary and secondary data. **(B)** Summary of food composition data reported by geographic region.

An inventory of 101 FCDBs was evaluated. Each database was characterized using 22 general database attributes, eight food and nutritional data attributes, and four criteria for FAIRness evaluation (Annex 1; Supplementary Tables S1–S5). Out of the 101 databases assessed, 73 (72%) FCDBs provide nutritional data for foods typically consumed within a single country, focusing primarily on local foods. In contrast, 28 (28%) of the databases compile nutritional data from food collected and consumed across multiple countries, often involving regions or neighboring countries. Among these international databases, 16 explicitly list the names of all countries contributing to the food data, whereas the remaining 12 adopt a broader international scope without specifying the countries included (Figure 3).

A note on database governance, 68 (67%) FCDBs were funded and managed by national governments, 24 (24%) FCDBs were transnational or international efforts, 16 (16%) FCBDs were managed by or associated with public universities, and 8 (8%) FCDBs were nonprofit initiatives. Among the nonprofit organizations of note, the Alliance of Bioversity-CIAT and International Food Policy Research Institute (IFPR), both CGIAR institutions, have supported the formation of several FCDBs including *The Periodic Table of Food Initiative* (PTFI), Biodiversity for Food and Nutrition (B4FN), and HarvestPlus’ *A Food Composition Table for Central and Eastern Uganda* (44, 45, 80).

The FCDBs were further stratified based on the origin of the data used. Eleven databases reported exclusively primary data, which consists of analytical data generated directly by the database entities; 42 relied only on secondary data, gathered from different secondary sources such as scientific articles or other FCDBs; 44 used a mix of primary and secondary data (Figure 3). Notably, countries hosting more than one database typically included both primary and secondary data. Likewise, databases from countries in Africa, Central America, and Eastern Europe predominantly contain secondary data (Figure 3).

We further analyzed the relationship between the data source (primary or secondary) and three key attributes: date of creation, number of food samples, and number of compounds per food across the 101 FCDBs. The oldest database in this analysis, the *Standard Tables of Food Composition in Japan* (46), dates back to 1950 In contrast, the most recent ones include the *Albanian Food Composition Table* (47) and *The PTFI* (PTFI Research Hub – Research Community and Resources for the Periodic Table of Food Initiative) published in 2022 and 2024, respectively.

**Figure 4:**
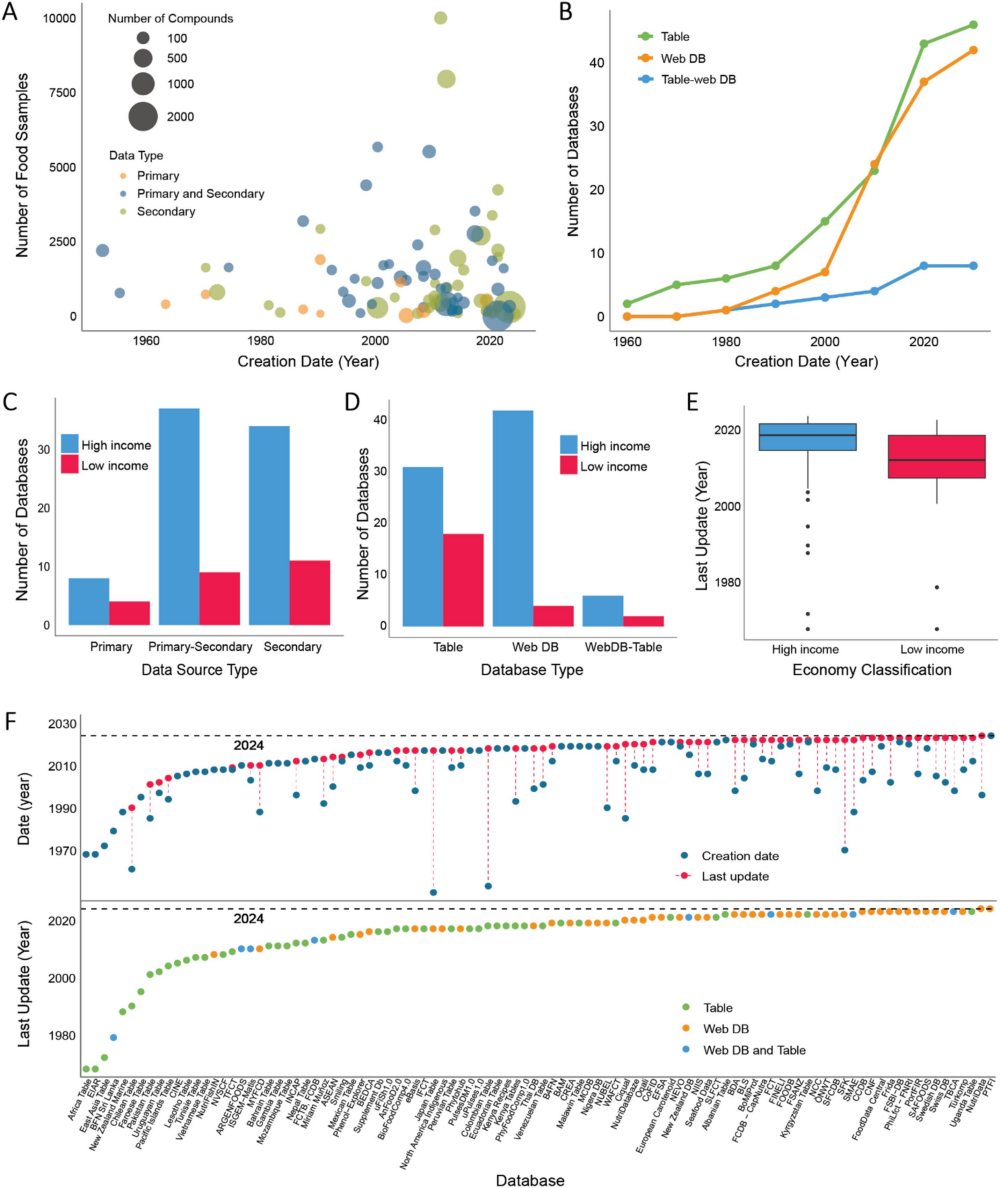
Overview of food composition databases (FCDBs) over time. **(A)** Bubble plot showing the relationship between the number of food samples covered in each database, the number of components reported, the type of data used (primary, secondary, or both), and the creation date. Eight outlier databases were excluded for better visualization. The size of each bubble represents the number of molecular components. **(B)** Line plot depicting the growth in the number of FCDBs created from 1950 to 2024 (year of review). Different colors represent the type of database interface: Table (green), Web interface (purple), and both Table and Web interface (blue). **(C)** Bar plot showing the number of FCDBs created by High-Income and Low-Income countries, categorized by database interface type (Table, Web, or both). **(D)** Bar plot representing the number of databases created by High-Income and Low-Income countries, categorized by data source type: Primary, Secondary, or both (Primary and Secondary). **(E)** Box plot comparing the distribution of the last update year for FCDBs created by High-Income and Low-Income countries. Countries grouped as High-income also include countries considered Upper-Middle income, and the category of Low-Income countries also includes countries classified as Lower-Middle income (42). **(F)** Upper graph: A timeline illustrating the creation dates (blue dots) and most recent updates (red dots) for the 97 FCDBs analyzed. Lower graph: A timeline displaying the last update dates for the 97 FCDBs, categorized by database interface: Table (green), Web interface (purple), or both Table and Web interface (blue).

The number of food samples listed across these databases varied significantly, ranging from a single food type in the *Bovine Milk Proteome Database* (48) and *The Milk Composition Database* (49) to 65,993 in the *L’observatoire de l’alimentation* database (*OQALI*), with an average of 2,523 food samples. Notably, 90% of the databases reported fewer than 4,000 food samples. Our analysis revealed that databases solely based on secondary data had the highest average number of food samples (range: 1 to 65,993; average: 3,614). Excluding outliers such as the *L’observatoire de l’alimentation* database (50), *Food and Nutrient Database* (51), and *The European Food Safety Authority Food Composition Data* (52), which report 65,993, 19,500, and 16,500 food samples respectively, the adjusted average for secondary data databases drops to 1,390. Mixed databases (primary and secondary data) ranged from 1 to 15,000 foods, averaging 1,988. Excluding outliers databases *FoodData Central* (10) and *German Nutrient Database* (53), which report 13,682 and 15,000 food samples respectively, the average number of food samples falls to 1,400. Databases with only primary data reported between 16 and 1,892 foods, averaging 488 (Figure 4A; outliers were excluded from graphical representation).

The count of compounds reported per database also showed considerable variation, from six in the *European Database of Carotenoids* (54) and *The Proximate Composition of New Zealand Marine Finfish and Shellfish* (55) to 70,926 in *FooDB* (56), with an average of 1,223 compounds. However, 90% of databases reported fewer than 550 components. According to our results, databases including primary data averaged the highest number of measured compounds (range: 6 to 24,721; average: 2313), but removing the outlier database *The PTFI,* which includes 24,721 compounds, reduces the average to 73. Mixed data databases varied from 15 to 70,926 compounds, averaging 1,756, but excluding the outlier FooDB drops the average to 147. Secondary databases ranged from 6 to 10,642 compounds, averaging 419, and removing the outlier *Bovine Milk Protein Database* with 10,642 compounds, adjusted the average to 181 (outlier databases were excluded from graphical representation - Figure 4A).

Analyzed FCDBs were formatted in different interfaces, 48 were web interfaces (48%), 45 were static tables (44%), and 8 included both web interfaces and static tables (8%). Originally, databases were primarily static tables. However, in the early 2000s, there was a clear shift toward web-based interfaces (Figure 4B). Interestingly, despite the growing popularity of DBs with web interfaces, table formats have continued to be a prevalent method for presenting nutritional information. Based on the World Bank economic classification of countries (42), 77% of FCDBs were created by High-Income countries, while 23% were created by Low-Income countries. As expected, FCDBs developed by High-Income countries primarily incorporate a web-based interface, while most of the FCDBs developed by Low-Income Countries rely on static tables (Figures 4C,D).

Although the number of table and web-based FCDBs has increased over time, their update frequencies show considerable variation (Figure 4F). Of all databases analyzed, 38 (39%) have never been updated. Among the remaining 59 databases, 11 (11%) have not received updates in the last decade. The update frequency also differs by database format; among table-based databases, 28 (61%) have never been updated and 7 (15%) were last updated over a decade ago. In contrast, among the 51 databases with web interfaces, only 10 (20%) have never been updated, with the majority (69%) updated in the last 5 years (Figure 4F). Additionally, the update frequency seems influenced by the economic classification of the country of creation of the database, with databases from High-Income countries generally showing more recent updates (Figure 4E).

### 4.3 Food and nutritional coverage across FCDBs

**Figure 5:**
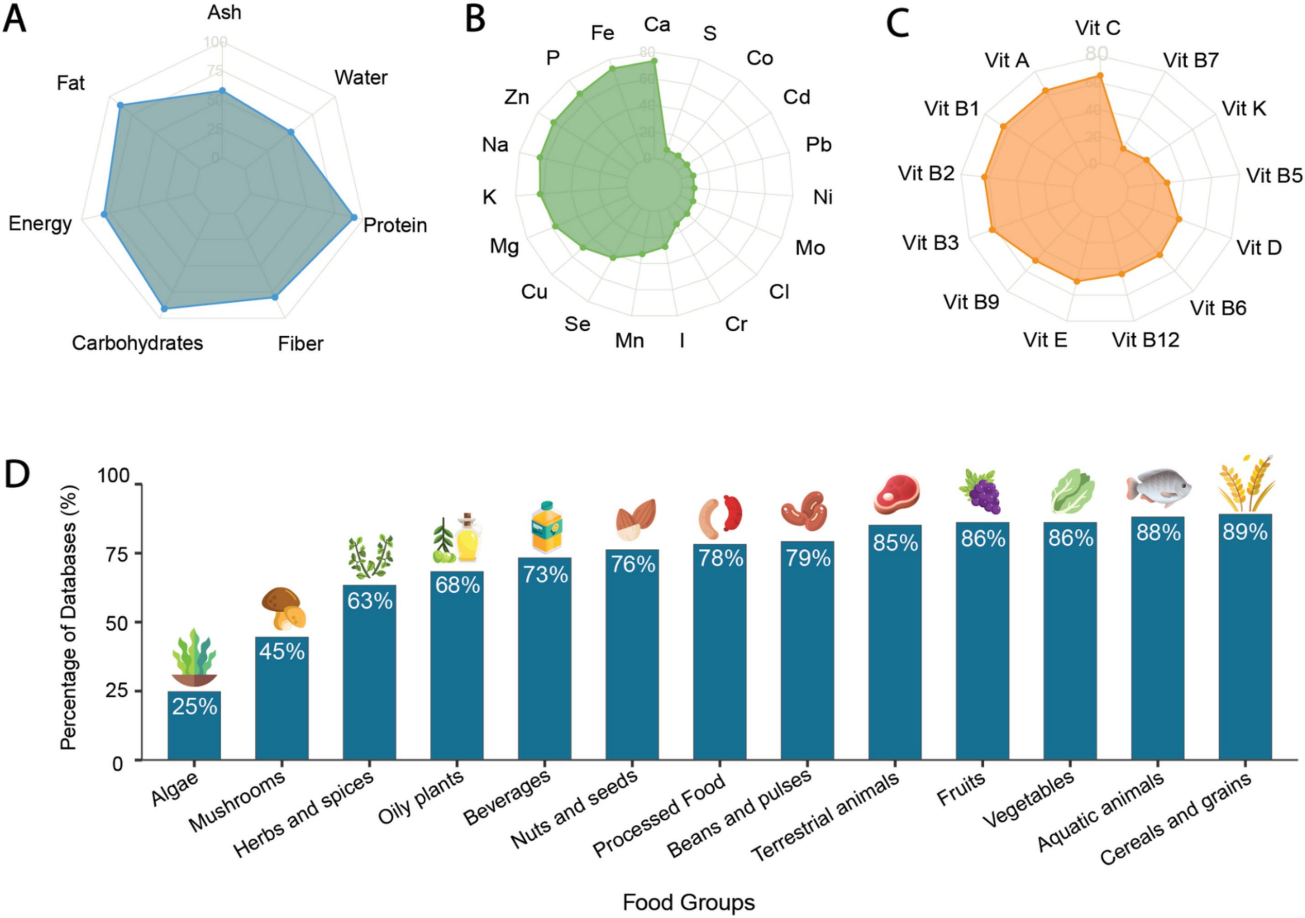
Coverage of food composition across food databases. **(A)** Report of proximate composition in Food Composition Databases (FCDBs). Chart axes represent different nutritional components each scale from 0 to 100. The plotted values indicate the number of databases that include data on each respective compound **(B)** Number of databases documenting minerals reported in at least 10 databases assessed in this study. **(C)** Number of databases documenting Vitamins reported in at least 10 databases assessed in this study **(D)** Presence of 13 food groups across the evaluated food composition databases, presented as the percentage of databases that include each food group.

Supplementary Table S2 summarizes the findings from the assessment of FCDBs, focusing on the food samples and components measured in each database, including both nutritional and bioactive compounds. We first examined the inclusion of commonly reported macro- and micronutrients, including proximate composition, minerals, and vitamins (Supplementary Table S7; Figures 5A–C). Among the evaluated FCDBs, 95 contained data on proximates or basic nutrients, 94 included minerals, and 91 covered vitamins. In contrast, only four specialized, compound-specific databases, such as *Phytohub* (57) and the *Bioactive Substances in Food Information Systems database* (58), focused exclusively on plant-based bioactive compounds.

Across all databases, a total of seven proximates were reported: water, ash, energy, fiber (i.e., crude and/ or dietary fiber), carbohydrates, fat, and protein. Among these, protein and fat were the most frequently included, reported in 90 and 91% of databases, respectively (Figure 5A). A total of 43 minerals were identified, but only 19 were reported in at least 10 databases (Figure 5B). Likewise, 18 vitamins were identified, with 12 of them being reported in at least 10 databases (Figure 5C; Supplementary Table S7). Specific bioactive compounds were also assessed across all databases. Notably, only 15% of the databases did not report any specific bioactive compounds beyond proximates, vitamins, and minerals. Of the 85% of databases reporting bioactives, the main groups of compounds were identified and reported as either compound class (e.g., fatty acids, amino acids, polyphenols, etc.) or specific compounds (e.g., cholesterol, tryptophan, beta-carotene, etc.; Supplementary Table S7). However, due to the diversity of compounds and nomenclature used, further comparison to evaluate the coverage of compounds across databases was not possible.

The landscape of edible biodiversity reported in food composition databases is extensive. However, in many cases, critical food-specific metadata and/or standardized food coding are absent. This lack of harmonization complicates the comparison of foods across databases beyond broad, culinary food group classifications. To address this limitation, we used 13 predefined food groups to assess and compare food coverage across all databases (Figure 5D). Our analysis revealed that *Aquatic animal products* and *Cereals and grain*s were the most common food groups, present in 89 and 88% of FCDBs, respectively. *Fruits and vegetables* were reported in 86% of databases, followed by *Terrestrial animals* reported in 85% of databases. Notably, infrequent coverage was observed for *Mushrooms* and *Algae*, which were included in only 45 and 25% of databases, respectively. Furthermore, the number of food groups represented in the FCDBs was analyzed (Supplementary Figure 1) revealing that 60% of databases included 10 or more of the 13 food groups. In contrast, 9% of databases reported only one single food group.

### 4.4 FAIRness of FCDBs

**Figure 6:**
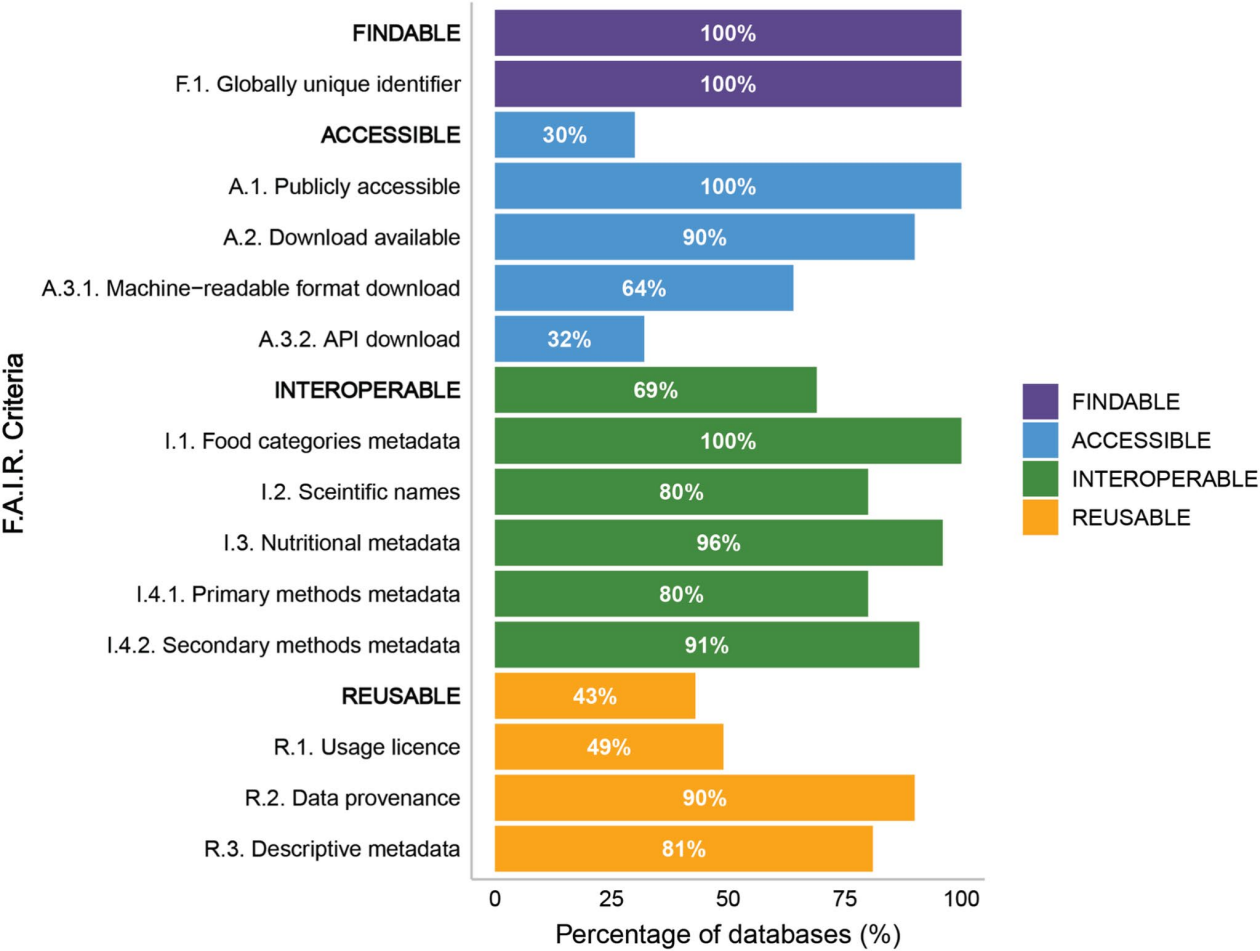
FAIR Data Principles criteria for Food Composition Databases (FCDBs). Bar graph illustrating the percentage of databases meeting the criteria for each FAIR principles Findable (purple), Accessible (blue), Interoperable (green), and Reusable (orange). For each category, the first item in uppercase represents the overall compliance percentage of databases with all the listed criteria under that principle, followed by the individual compliance percentages for specific criteria within the category. Detailed information on the criteria used to evaluate the FAIR data principles is provided in Supplementary Table S3.

Following the criteria outlined in this manuscript for evaluating the FAIRness of global nutritional databases (Supplementary Table S3), we established the percentage of databases that adhere to FAIR principles of being Findable, Accessible, Interoperable, and Reusable (Figure 6) (34). Based on our findings, every database included in this study met the findable criteria, meaning that each one is assigned a persistent identifier. This identifier, which can be a Uniform Resource Locator (URL) or a Digital Object Identifier (DOI), ensures that the databases can be accurately identified by both human users and computers. However, two databases were excluded in the first step because their URLs were broken (i.e., access yielded a 404 error: page not found).

Our analysis revealed that 100% of the databases adhere to the criteria of being publicly accessible through either open or paid access. However, a closer examination of the access modes reveals limitations in access. Specifically, 10% of the databases did not have download availability, thereby making it impossible to interact with the raw data. Among the databases that did allow downloads, only 64% support the downloading of machine-readable files, underscoring a limitation in the versatility of data formats provided. Additionally, the option to download data via an API is scarce with only 32% of databases offering this path for automated data retrieval. Collectively, only 30% of databases met all accessibility criteria evaluated.

Evaluation of the interoperability of FCDBs showed that food classification systems were consistently reported across all databases. Food components metadata were included in 96% of the databases, and metadata for methods used in secondary databases was present in 91% of cases. The areas with the least compliance were the inclusion of scientific names for the foods analyzed, which was found in 80% of FCDBs, and the inclusion of metadata related to the analytical methods used in primary databases, which was present in 80% of the databases. Overall, 69% of the FCDBs met all the criteria for interoperability.

The assessment of data reusability revealed high compliance in reporting data provenance (90%) and providing descriptive general metadata (81%). However, the most commonly missing criterion was the lack of a licensing statement regarding the use of the data, which was present in only 49% of the databases. Overall, only 43% of FCDBs met all the criteria for data reusability.

Overall, the analysis of the FAIR principles indicates that, although several FAIR criteria are being implemented by national and international FCDBs, significant gaps in adoption remain. Notably, only 17 databases (17%) satisfied all 13 FAIR criteria evaluated in this integrative review. All of these FCDBs are accessible through web interfaces and are associated with High-Income countries (Table 1).

**Table 1:** Food composition databases that met all the FAIR criteria analyzed in this integrative review.

Database	Creation date	Last update	Creation country	Database type
ANSES-CIQUAL	1985	2020	France*	Web DB
Canadian Nutrient File (CNF)	2007	2023	Canada*	Web DB
Composition of Foods Integrated Dataset (CoFID)	2008	2021	United Kingdom*	Web DB
Czech Food Composition Database (NutriDatabaze)	2010	2020	Czech Republic*	Web DB
Dutch Food Composition Database (NEVO)	2019	2021	Netherlands*	Web DB
Finnish National Food Composition Database (FINELI)	2019	2022	Finland*	Web DB
Frida Food Data	2002	2023	Denmark*	Web DB
Food Composition Database for Epidemiological Studies in Italy (BDA)	1998	2022	Italy*	Web DB
FOODB	2020	2022	Canada*	Web DB
Food Data Central	2019	2023	United States*	Web DB
The Periodic Table of Food Initiative (PTFI)	2024	2024	United States*, International	Web DB
New Zealand Food Composition Database	2015	2021	New Zealand*	Web DB; Table
Spanish Food Composition Database (BEDCA)	2010	2016	Spain*	Web DB
Tabelle di Composizione Degli Alimenti (CREA)	2019	2019	Italy*	Web DB
The Norwegian Food Composition Table (FCT)	2012	2022	Norway*	Web DB; Table
The Swiss Food Composition Database	2002	2023	Switzerland*	Web DB
Turkish Food Composition Database (Türkomp)	2008	2023	Turkey*	Web DB

*High-Income Economy based on World Bank Data (42).

It is important to note that while we evaluated the FAIRness of FCDBs, we did not assess the accuracy of food composition data. The FAIRness criteria for interoperability only indicated if analytical methods—used in the collection of food composition data—were reported. We did not evaluate the validity of the analytical methods used to generate primary data. These factors are crucial for assessing the reliability and accuracy of nutritional data but fall outside the scope of this review.

## 5 Discussion

In the era of digital innovation, food data quality and utility are key. Yet our integrative review of 101 FCDBs revealed that global efforts often have inadequate coverage of both foods and food components in the world’s food supply. We found a skewed geographic distribution, with North America, Europe, and Asia having the highest representation with more than 15 databases per continent. Even where FCDBs are plentiful there is still an opportunity to improve the coverage of both foods and food components. Our search revealed, on average, FCBDs contained 2,523 food samples and 1,206 food components; however, only 38 components (i.e., proximate composition, select minerals and vitamins) were found to be common among at least 10 databases. To fill in gaps in both the number of foods and components, FCDBs often recycle secondary data from existing databases; we found 85% of databases used at least some secondary data. Food composition and the composition of diets can evolve over time due to environmental, economic, and social dynamics. Yet, 39% of FCDBs have yet to be updated, speaking to the opportunity for regions to renew their understanding of their own food supply. However, we recognize that countries’ capacity to update their food composition databases is very much dependent upon economic status. For example, only 12 databases that meet the FAIR criteria are web-based FCDBs, and they are maintained by high and upper-middle-income economies. Overall, we recommend global FCDBs work toward expanded coverage of foods and components, unified methods of analysis, and enhanced metadata and FAIR data adherence, all to improve scope and harmonization across FCBDs globally. Through our assessment of the profile of food composition databases, we found emergent opportunities to improve the quality and usefulness of FCDB content and propose the following key recommendations for improvement:

### 5.1 Emergent opportunities

1. Geographic distribution of food composition databases
  Through this integrative review of the state of FCDBs, we found that many countries around the world do not produce or maintain a national FCDB containing nutritional information about their locally consumed foods. Further, where national databases exist, we found an absence of primary analytical data, particularly in regions like Central America, East Asia, and across the continent of Africa. The discrepancy in data availability is often correlated with economic classification; typically, high-income countries not only include more primary data but also frequently update their databases and utilize web interfaces. The disparity between high and low-income countries’ capacity to generate data on their own food composition data has downstream implications for dietary guidelines, food and agriculture policymaking, and ultimately human and planetary health outcomes. On the continent of Africa, the absence of national FCDBs with primary data further complicates efforts to devise optimal dietary improvement strategies to combat the prevalence of malnutrition and chronic and hidden hunger (18, 59). In Africa, Southeast Asia, and beyond, opportunities exist to apply sustainable food-based approaches to address micronutrient deficiencies through biodiverse dietary recommendations powered by high-quality, primary food composition data (60–62).
2. Prevalence of primary versus secondary data
  The reliance on secondary data, where primary data are unavailable, poses considerable risk. Secondary data do not accurately reflect the current local food supply in coverage of foods nor in food components. Primary data compiled into secondary databases from other geographies should not be used as a one-to-one swap (37), especially in the absence of metadata and FAIR data standards of interoperability. Further, the use of secondary data often leads to instances where data do not reflect recent advances in analytical methods, plant breeding or agricultural advancements, or changes in food processing methods (10, 63, 64). Data inaccuracies are also propagated when FCDB data are used to describe composite meals. Ingredient substitutions with foods bearing similar common names or with foods grown in different geographies often present a multitude of confounders that lead to dietary assessment errors in human nutrition studies (20). The information obtained from current dietary assessment tools carries an inherent bias that is rooted in their retrospective nature. This bias is further amplified by the inaccurate compositional analyses of the habitual diets of individuals which encumbers the understanding of robust diet-health associations (65).
  Additionally, in the era of digital innovation, AI tools using large language models trained on recycled secondary data will undoubtedly result in misleading conclusions termed artificial hallucinations (11, 66), particularly when certain geographies are overrepresented in the data. Knowing that only 15% of databases were solely powered by primary food composition data strongly points to the need for democratized tools to generate primary data to support AI applications.
3. Number of food components and coverage
  Since the 1990s, food biomolecular diversity among databases has increased slightly, but most databases are still limited to under 100 food components measured. Our findings demonstrate that FCDBs report on average 1,206 food components (ranging from 6 to 70,926). However, 90% of databases reported fewer than 550 components with known bioactivity. Moreover, the reality is that only 38 components were found to be common among 10 databases with proximate composition, minerals, and vitamins dominating the landscape. We observed a slight trend deviating from these few components since the 1990s, but there is much work to be done to further uncover the nutritional dark matter of food, particularly when the chemical complexity in our diet ranges from an estimated 26,000 to 49,000 distinct biomolecules (67).
  Two noteworthy outliers stood out among the rest in addressing this unknown chemical space. The Bovine Milk Protein Database and FooDB with 10,642 compounds and 70,926 compounds, respectively, are unique in the sheer number of compounds reported although secondary data was included. The PTFI was notable for reporting a large number of compounds (i.e., 24,721 compounds) generated as high-confidence, primary data. This is a welcome development in the FCDB space since, through our review, comparability of specific compounds beyond proximate composition, nutrients, and vitamins was an insurmountable limitation. Biomolecular diversity stands out as a major limitation of data comparability.
  These challenges highlight the urgent need for improved standardization and reproducibility in generating primary data. Beyond the most commonly measured nutrients, there are limited globally accepted standardized methods to evaluate food’s diversity of bioactive molecules. Additionally, analytical limitations can arise from the need for diverse instrumentation tailored to each type of biomolecule. The complexity of the food matrix drives accessibility challenges of costly, time-consuming, and low-throughput extraction and analytical methods (1). To address these challenges, international food composition databases and ontologies are emerging, establishing improved data standards, particularly for food components.
4. Number of food samples and coverage
  Our search revealed, on average, FCBDs contained just 2,576 food samples underscoring the opportunity to improve food coverage in databases globally. We identified some outlier databases such as OQALI which includes a large number of food samples derived from secondary data. By contrast, the Malaysian Food Composition Database, reported only self-generated, primary data on 1,892 foods. Overall, databases with primary data average only 488 food samples. Food samples in our review are defined as food items. Food items include whole, raw foods, and minimally processed foods, but also multi-ingredient meals and processed and packaged food items, etc. A large count of food samples is not necessarily indicative of edible species diversity, and in most cases, food species-specific metadata were missing to make that determination.
  On the topic of food coverage, only 20% of the databases reviewed encompassed all 13 culinary food groups. Yet, we additionally found the food group categorization often lacked standardization. We found ambiguity in classification of certain food classes like mushrooms and insects, with very few databases including metadata to support more accurate food group classification. The best examples of food-specific metadata appear in EuroFIR FoodEXplorer, FAO/INFOODS, and USDA FDC databases.
  By our estimation, INFOODs and USDA FDC (i.e., Foundation Foods and Standard Reference Legacy) combined report approximately 119,922 food samples but only 767 diverse species (18, 23, 40). By contrast, the PTFI, when leading a search to determine gaps in species coverage, created an inspiring list of 1,650 species of high priority in need of biomolecular exploration (23). In terms of both the bio- and chemodiversity of food, these initiatives represent a small fraction (i.e., ~5%) of the estimated 35,000 edible plant (22, 24, 68), animal, insect (25, 69), and fungal (70, 71) species worldwide. This leaves 95% of named edible species yet to be explored.
5. Frequency of update
  In addition to improving the breadth of food and nutritional data, measuring food composition consistently and over time can provide a basis for identifying drivers of food quality, such as genetic variation, agricultural practices, climate, food processing and preparation, and consumer preferences (4, 11). Yet, 59 out of the 101 databases and tables we surveyed have not published updated food composition data in the last decade. The update frequency among databases was better than data tables, with a majority (69%) of web-based FCDBs having been updated in the last 5 years. However, the opportunity for more relevant food composition data remains, particularly to keep pace with a rapidly changing climate.
6. Adherence to FAIR data management and stewardship
  Harmonizing food composition data is foundational in ensuring consistency, accuracy, interoperability, and traceability across various food composition databases and sources. Currently, there is no standard for assessing the data quality of FCDBs (8), but the *FAIR Guiding Principles for scientific data management and stewardship provide an initial framework* to understand how food data might be structured and utilized within these FCBDs. The FAIR principles also point to a significant need for enhancing the homogenization and comparability of FCDBs. Several challenges related to the findability and accessibility of FCDBs were identified such as broken URLs. Notably, some of the databases surveyed in this integrative review were embedded in food composition data indexes that act as repositories for FCDBs. These embedded data sources were not independently findable through a web search and could only be located by visually scanning food composition data index web pages. Although most databases are publicly accessible, the formats available often hinder effective interaction with the data, as many only provide PDF-based food tables and web-based interfaces lack APIs to facilitate data exchange.
  Interoperability is still a critical challenge according to our results underscoring the need for clearer descriptions of analytical methods and scientific nomenclature for food components, which are crucial for enhancing data reliability and comparability. The analytical methodologies used in populating these databases often vary by country or even institution, as does the naming of foods and nutritional components making the comparisons between databases challenging (60). While there has been widespread adoption of FAO/INFOODS tag names and EuroFIR thesauri for food components, facilitating some standardization in language across databases, there remain significant gaps in the standardization of these components across other databases globally (40, 72).
  Noteworthy, most FCDBs adequately describe their in-house metadata, which not only supports the potential for reusability but also facilitates deeper analysis and broader application in diverse research contexts. Efforts to harmonize procedures for better data comparability and interchange, such as the FCDBs of FAO/INFOODS and EuroFIR FoodEXplorer and independent ontologies like the Food Ontology (FoodON) have aimed to address these challenges (35). However, wider adoption among other FCDBs has been slow, hindering the effectiveness of data interoperability (11). Furthermore, the description of usage rights attached to the data and metadata often remain vague, which can significantly restrict the potential for reusing and sharing data across studies and applications.
  The FAIRness of FCDBs is crucial for analyzing and comparing data from different databases considering the criteria used in each country, the diverse objectives pursued by each project, and the intended users (11, 73). Studies from the late 1990s suggest that nutrient intake estimations from the same diet can vary by as much as 20–45%, depending on the database used, owing to systematic and random errors that include discrepancies in naming, terminology, and calculation methods (74). It is worth noting that the FAIRness of the analyzed databases is highly related to the income classification of the country that developed them. This underscores the need for greater support, resources, and guidelines to ensure more consistent and accurate comparisons across databases globally.

## 6 Recommendations

The depth of our understanding of food composition significantly influences our ability to develop sustainable diets and improve nutritional outcomes. Recognizing the various challenges involved in the collection and dissemination of quality, standardized, and well-organized food composition data, credit is given to the significant efforts that have been made. These efforts complement existing strategies aimed at enhancing dietary quality. Overall, we recommend the following actions for advancing the utility of food composition data for diverse types of users. We recommend efforts be made in five key areas:

1. Broaden database content and frequency of update through globally coordinated and place-based approaches
  To accurately profile the vast diversity of modern diets and the global edible biodiversity more broadly, it is essential to expand the range of foods and components included in food composition databases, including those that reflect diverse cultures, agricultural practices, and geographies. Ensuring regular updates is crucial for maintaining the accuracy and relevance of the data, particularly in a changing climate and with changes in land use, agriculture, and food systems. Advancements in technology such as foodomics approaches further warrant the continuous update of food composition databases as new methodologies are developed to more precisely profile food composition. While resources remain a constraint in profiling a wide range of foods and biomolecules in FCDBs as well as their update frequency, a globally coordinated approach among countries would support economies of scale and enable countries to benefit from learnings globally. Such a globally coordinated approach should include place-based efforts representative of local food systems including underutilized crops as well as novel crops currently under development. Further, web-based platforms, known for their flexibility and ease of access, can significantly facilitate these updates and allow for monitoring of shifts in food component data over time.
2. Harmonize data and enhance quality using standardized approaches and metadata
  To achieve a comprehensive and cohesive approach to food data collection, we recommend a minimum, globally agreed upon, set of food components generated using standardized methodologies for inclusion in FCDBs to enhance interoperability globally and provide evidence on the world’s food supply at scale. Standardized methods, including foodomics approaches (1, 75) to comprehensively profile food components using both targeted methods and untargeted methods (i.e., techniques to measure unknown compounds with relative quantitation), is essential to expand our understanding of the vast, unknown “dark matter” of food. Complementing these untargeted analytical techniques with relative quantification of compounds of importance for human and planetary health can further enhance the functionality of these data. Yet even more fundamentally, to add context to food data, we recommend the inclusion of metadata. Accurate descriptions of analytical methods through the use of metadata and data dictionaries, including nomenclature for foods and components, will be a hallmark of this next era of digital innovation.
3. Incorporate FAIR principles and ethical governance and stewardship
  Incorporating Findable, Accessible, Interoperable, and Reusable (FAIR) principles within FCDBs will enhance their utility. This involves improving the findability and accessibility of FCDBs by maintaining functional links and incorporating APIs and machine-readable formats, which simplifies data integration and usage. Furthermore, implementing clear and standardized usage licenses is essential to promote the reusability of FCDB data across various studies and applications, ensuring that data usage rights are well-defined and communicated. Ultimately, leveraging existing ontologies and building out new food systems-focused thesauri will enhance the interoperability of food composition data in this new era of digital innovation (76, 77).
  Beyond data-centric FAIR principles, there is an emerging awareness of data governance, stewardship, and ethics globally. Because food composition data and associated metadata are effectively digital sequence information (DSI), food composition repositories should likewise be tasked with adhering to access and benefits sharing modalities governing the use of other data derived from genetic resources. In light of the outcome of negotiations at the United Nations Conference of the Parties Convention on Biological Diversity (CDB COP16), databases compilers, and by extension FCDBs, have a call to action to infuse FAIR, CARE, and TRUST principles into their data governance policies. CARE, or the *CARE Principles for Indigenous Data Governance,* is an acronym meaning Collective Benefit, Authority to Control, Responsibility, and Ethics (78). CARE principles promote data sovereignty ensuring responsible data collection, accreditation, and equitable data reuse. Whereas TRUST is an acronym for Transparency, Responsibility, User-focus, Sustainability, and Technology (79). TRUST principles foster sustainable governance of digital repositories by promoting reliable and secure infrastructure over the long term. Integration of the complementary principles FAIR, CARE, and TRUST will encourage database managers to honor both people and purpose in the stewardship of food data. Food composition databases potentially contain vast amounts of digital Indigenous data and traditional knowledge, and therefore, have a responsibility to steward these datasets ensuring that the data is safeguarded from historical inequities.
4. Strengthening capacity for generating and applying food composition data across food systems
  While food composition data have historically been utilized for nutritional assessments and by nutritionists and dieticians, there are increasing opportunities to apply food composition data across food systems by diverse stakeholders including farmers, producers, crop breeders, and agricultural researchers, but also food scientists, food manufacturers, chefs, consumers, and other diverse users. For example, for these diverse users to know how to apply food data, there is a need for capacity strengthening. In addition, as novel approaches for generating food data such as foodomics provide emerging opportunities, there is a need to provide capacity strengthening to scientists globally on utilizing these novel technologies. Capacity strengthening in the form of technology transfer should not only bolster the technical aspects of FCDBs but also enhance their applicability and use in diverse cultural contexts.
5. Prioritize investment to develop and maintaining FCDBs
  High-quality FCDBs require notable resources. There is a pronounced need for increased investment to support the creation of accurate, accessible, and culturally relevant FCDBs, particularly in countries with limited resources and capacities. As food data are beneficials for those across food and health systems, there is a need to prioritize investment in FCDB infrastructure for diverse users across food and health sectors. Likewise, such efforts should be equitable globally. In an increasingly globalized food system, it is essential for high-income countries to support FCDB efforts in low-and middle-income countries from where they often procure food.

## 7 Conclusion

Food composition data are essential for informing solutions and decision making to today’s human and planetary health crises, including biodiversity loss, food insecurity, and diet-related chronic diseases. Despite the critical role of FCDBs, this integrative review reveals a significant opportunity to improve the coverage, structure, and comparability of data on food components. Many FCDBs include data on only a few foods and components, with a small subset consistently reported across databases. In addition, there is a high level of reliance on secondary data and a widespread use of static tables for representing the data. These challenges underscore major gaps in the availability of robust and updated nutritional data, limiting the relevance of these databases in specific cultural and geographic contexts.

Data stewardship guidelines, like the FAIR data principles, demonstrate areas for improvement and progress. While all FCDBs meet some criteria, such as Findability, only a few fully adhere to all FAIR principles, with a clear need to improve machine-readability and data reusability. Notably, high-income countries are frequent adopters of web-based interfaces, frequently updated platforms, and FAIR principles compared to middle-low-income countries. Encouragingly, some efforts have arisen to address these challenges, resulting in several international food composition databases with improved data standards, especially for food components. FCDBs like FoodData Central and FoodDB have set high standards for data quality and breadth, respectively, while newer projects, such as the PTFI, contribute innovative analytical approaches, meta-data and data harmonization.

To overcome the current challenges in FCDBs, we recommend: (i) broadening the coverage of foods and bioactive compounds included in FCDBs to better represent global dietary diversity, (ii) establishing standardized methods for data generation, curation, and reporting to enhance interoperability, (iii) comprehensively implementing FAIR principles, including higher resolution metadata, to improve data accessibility and usability, and (iv) increasing investments in capacity building and technological infrastructure, particularly in resource-limited regions. Strengthening FCDBs through these strategies will significantly enhance their utility for policymakers, researchers, and practitioners. This advancement will support the development of evidence-based nutritional profiling and guidelines, foster biodiversity conservation, and contribute to more sustainable diets and equitable food systems that promote human and planetary health.

## Data Availability

The datasets presented in this study can be found in online repositories. The names of the repository/repositories and accession number(s) can be found in the article/Supplementary material.
