# Supplementary figures and images for: The state of food composition databases: data attributes and FAIR data harmonization in the era of digital innovation
Source: Front Nutr. 2025 Mar 19;12:1552367. doi: 10.3389/fnut.2025.1552367 (PMC11974508; doi:10.3389/fnut.2025.1552367)

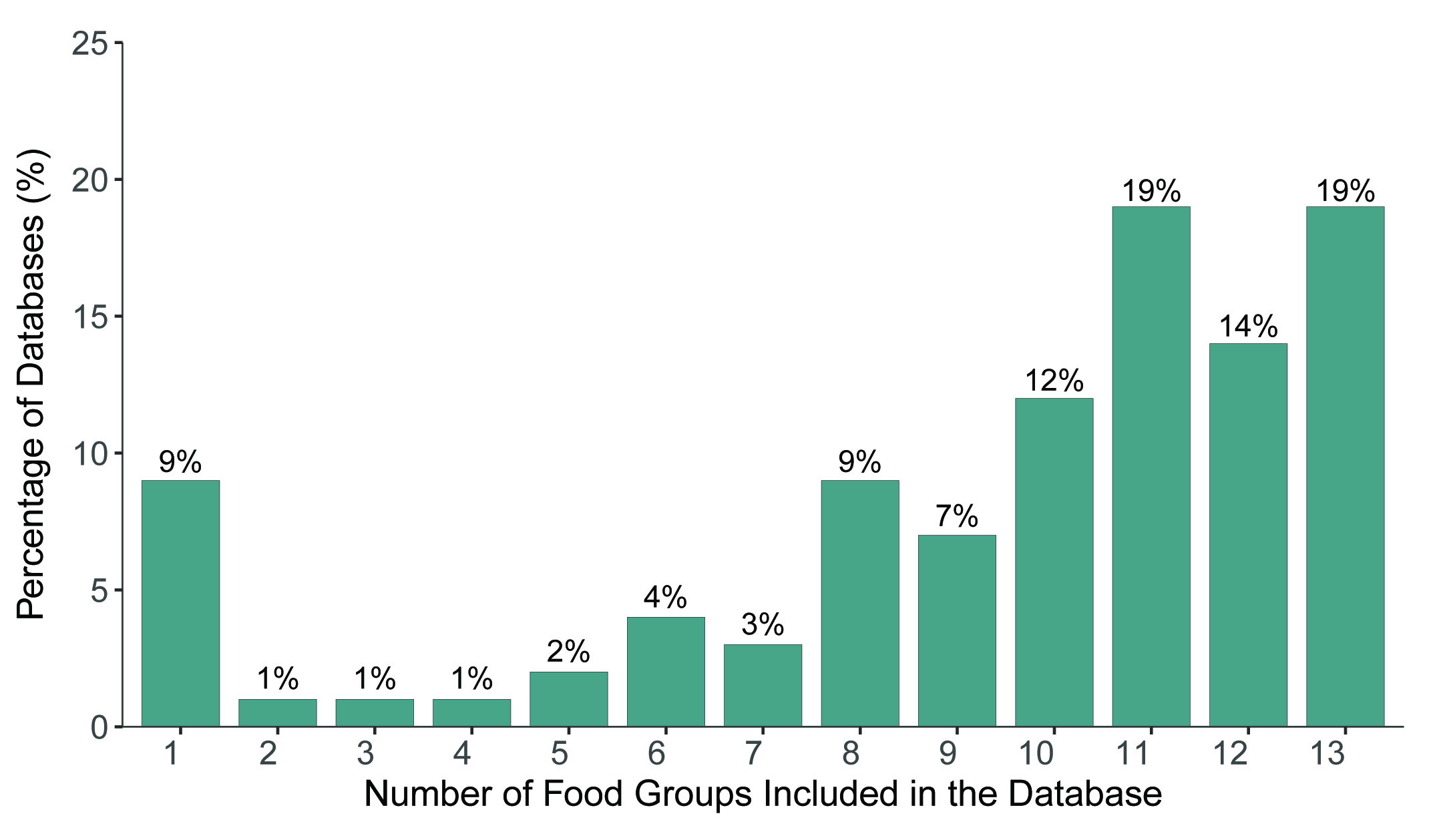

Supplement: Supplementary file 1 [file Image_1.TIF]
